# Multiple Sclerosis Progression Discussion Tool Usability and Usefulness in Clinical Practice: Cross-sectional, Web-Based Survey

**DOI:** 10.2196/29558

**Published:** 2021-10-06

**Authors:** Tjalf Ziemssen, Gavin Giovannoni, Enrique Alvarez, Virender Bhan, Carrie Hersh, Olaf Hoffmann, Celia Oreja-Guevara, Rene R Robles-Cedeño, Maria Trojano, Patrick Vermersch, Pamela Dobay, Mudeer Khwaja, Bianca Stadler, Benedict Rauser, Thomas Hach, Daniela Piani-Meier, Jason Burton

**Affiliations:** 1 Center of Clinical Neuroscience Neurological University Clinic Carl-Gustav Carus Dresden Germany; 2 Blizard Institute Barts and the London School of Medicine and Dentistry Queen Mary University of London London United Kingdom; 3 University of Colorado School of Medicine Aurora, CO United States; 4 University of British Columbia Vancouver, BC Canada; 5 Cleveland Clinic Lou Ruvo Center for Brain Health Las Vegas, NV United States; 6 Department of Neurology St Josefs-Krankenhaus Potsdam Germany; 7 Department of Neurology Hospital Clínico San Carlos Madrid Spain; 8 Department of Neurology Girona Neuroimmunology & Multiple Sclerosis Unit Dr. Josep Trueta University Hospital & Santa Caterina Hospital Girona Spain; 9 Department of Basic Medical Sciences Neuroscience and Sense Organs, University of Bari Bari Italy; 10 University of Lille, INSERM U1172 Lille Neuroscience and Cognition, CHU Lille, FHU Precise Lille France; 11 Real World Evidence Solutions, IQVIA AG Basel Switzerland; 12 Novartis Pharma AG Basel Switzerland; 13 Centre for Neuromuscular and Neurological Disorders Western Australian Neuroscience Research Institute The University of Western Australia Perth Australia

**Keywords:** multiple sclerosis, relapsing remitting multiple sclerosis, secondary progressive multiple sclerosis, transition, progression, digital, usability

## Abstract

**Background:**

A digital tool, Multiple Sclerosis Progression Discussion Tool (MSProDiscuss), was developed to facilitate discussions between health care professionals (HCPs) and patients in evaluating early, subtle signs of multiple sclerosis (MS) disease progression.

**Objective:**

The aim of this study is to report the findings on the usability and usefulness of MSProDiscuss in a real-world clinical setting.

**Methods:**

In this cross-sectional, web-based survey, HCPs across 34 countries completed an *initial* individual questionnaire (comprising 7 questions on comprehensibility, usability, and usefulness after using MSProDiscuss during each patient consultation) and a *final* questionnaire (comprising 13 questions on comprehensibility, usability, usefulness, and integration and adoption into clinical practice to capture the HCPs’ overall experience of using the tool). The responses were provided on a 5-point Likert scale. All analyses were descriptive, and no statistical comparisons were made.

**Results:**

In total, 301 HCPs tested the tool in 6974 people with MS, of whom 77% (5370/6974) had relapsing-remitting MS, including those suspected to be transitioning to secondary progressive MS. The time taken to complete MSProDiscuss was reported to be in the range of 1 to 4 minutes in 97.3% (6786/6974; *initial*) to 98.2% (269/274; *final*) of the cases. In 93.54% (6524/6974; *initial*) to 97.1% (266/274; *final*) of the cases, the HCPs agreed (4 or 5 on the Likert scale) that patients were able to comprehend the questions in the tool. The HCPs were willing to use the tool again in the same patient, 90.47% (6310/6974; *initial*) of the cases. The HCPs reported MSProDiscuss to be useful in discussing MS symptoms and their impact on daily activities (6121/6974, 87.76% *initial* and 252/274, 92% *final*) and cognitive function (5482/6974, 78.61% *initial* and 271/274, 79.2% *final*), as well as in discussing progression in general (6102/6974, 87.49% *initial* and 246/274, 89.8% *final*). While completing the final questionnaire, 94.9% (260/274) of the HCPs agreed that the questions were similar to those asked in regular consultation, and the tool helped to better understand the impact of MS symptoms on daily activities (249/274, 90.9%) and cognitive function (220/274, 80.3%). Overall, 92% (252/274) of the HCPs reported that they would recommend MSProDiscuss to a colleague, and 85.8% (235/274) were willing to integrate it into their clinical practice.

**Conclusions:**

MSProDiscuss is a usable and useful tool to facilitate a physician-patient discussion on MS disease progression in daily clinical practice. Most of the HCPs agreed that the tool is easy to use and were willing to integrate MSProDiscuss into their daily clinical practice.

## Introduction

### Background

Multiple sclerosis (MS) is a chronic debilitating disease of the central nervous system that primarily affects young adults [1]. In most of the patients, the disease evolves as a continuum from the relapsing-remitting phase (known as relapsing-remitting MS [RRMS]) to the secondary progressive phase (known as secondary progressive MS [SPMS]) [2,3]. It is challenging to define this transition from RRMS to SPMS because of the lack of a clear consensus on the diagnostic criteria and the absence of reliable biomarkers of disease progression [4]. This delay in SPMS diagnosis may affect long-term prognosis and treatment decision-making [5]. Previous research has confirmed an unmet need for a tool to facilitate systematic assessment of the early signs of progression to SPMS in routine clinical practice [6].

The Multiple Sclerosis Progression Discussion Tool (MSProDiscuss) is a digital tool for use by health care professionals (HCPs) in clinical practice to raise awareness of the risk of progression from RRMS to SPMS through a structured interaction between HCPs and patients. MSProDiscuss aims to help physicians, in dialog with patients, to evaluate early, subtle signs suggestive of MS disease progression [7]. The tool is based on a set of weighted questions that collect structured information on disease activity (relapses or magnetic resonance imaging activity), symptoms, and impact of the patient’s overall symptoms on daily living in the previous 6 months. The tool is completed by the physician during a routine physician-patient interaction. On completion, the tool generates a traffic light output that represents the probability of progression. Green indicates patients who are unlikely to be showing signs of progression, yellow suggests that such signs may be present, and red identifies patients who are very likely showing signs of progression. MSProDiscuss was developed in several phases using a rigorous mixed methods approach. This approach included quantitative analysis of data from a large observational study in patients diagnosed with RRMS and SPMS and qualitative research with MS neurologists and patients with MS. The development of MSProDiscuss involved an iterative feedback process and validation stages [6-8]. The feedback received from patients and physicians was integrated into the next iteration throughout the development phases of MSProDiscuss. The final tool was pilot tested in a separate validation study with clinicians (N=20, from the United States, Germany, and Canada) in a real-world setting, and it demonstrated high sensitivity and specificity to differentiate between patients with RRMS and those with SPMS. The tool also demonstrated evidence of construct validity, suggesting that the items included are relevant in assessing early signs of progression, and the HCPs supported the implementation and usefulness of the tool for clinical practice [8,9]. MSProDiscuss is part of several noninterventional longitudinal studies to further assess changes in the level of progression over the long term [10,11]. MSProDiscuss has been released for use in clinical practice, and the final validated tool can be accessed on the web [12] and on the Neuro-Compass medical education resource website [13].

### Objective

We conducted a separate usability testing study to assess the performance of MSProDiscuss on a larger scale, involving HCPs from different geographies and health care systems, with the aims to further test the (1) usability of tool in daily clinical practice, and comprehensibility of items included: (2) usefulness of the tool to assist patient-physician discussion on MS disease progression, (3) feasibility, ease, and willingness of HCPs to integrate MSProDiscuss into their routine clinical practice, and (4) insights gathered from HCPs on areas of improvement on the tool’s usefulness. In this paper, we report the findings from physicians on the usability and usefulness of MSProDiscuss while discussing disease progression with patients in a real-world setting.

## Methods

### Study Design

This was a multinational, cross-sectional, and noninterventional study surveying HCPs, including MS specialists, general neurologists, or others as indicated by the self-identification choices provided to the participants. The HCPs responsible for the diagnosis, management, and care, or those in charge of symptom evaluation and control, of at least five patients with MS per week in their daily clinical practice were included in the survey. The HCPs were invited by local representatives of the study sponsor, Novartis, to participate in a web-based survey conducted between July 2019 and December 2019 in 34 countries across North America, Europe, Asia, South America, Africa, and Australia. No ethics approval was required because this was a survey. Nevertheless, all respondents were offered detailed information about the survey method, and they provided written consent before participating.

### Survey Methodology

**Figure 1:**
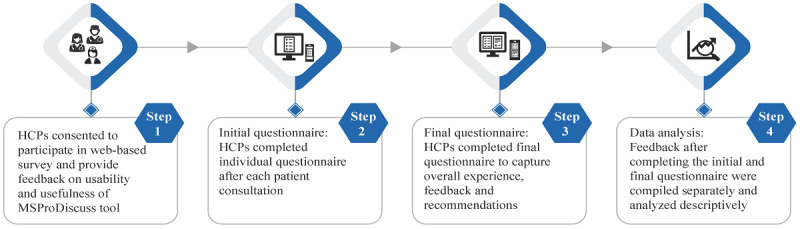
Steps of the MSProDiscuss usability test survey. HCP: health care professional; MSProDiscuss: Multiple Sclerosis Progression Discussion Tool.

The survey was carried out by Real World Evidence Solutions (IQVIA AG). A web-based survey link was sent to the HCPs who consented to participate in the study. The survey consisted of 2 questionnaires to gather feedback on the understanding, usefulness, usability, integration, and adoption of MSProDiscuss in daily clinical practice. The questionnaires were developed based on feedback from HCPs during the tool development phase and according to the principles of implementation science, and inputs provided by the sponsor’s medical team and by researchers from Real World Evidence Solutions were included. The initial translations of the questionnaires were provided by IQVIA, which were further evaluated and modified as necessary by the local Novartis medical teams. The HCPs used MSProDiscuss to assess what they felt was a broad range of people with MS, excluding those with clinically isolated syndrome and primary progressive MS. The survey methodology is illustrated in Figure 1.

Each HCP was asked to fill two types of questionnaires (Multimedia Appendix 1), with their responses collected on a 5-point Likert scale. The HCPs filled an initial questionnaire after each instance of using MSProDiscuss during a face-to-face individual patient consultation. This initial questionnaire included 7 questions to collect feedback on the time needed to complete MSProDiscuss during a routine clinical visit, comprehensibility of the questions in the tool, and the overall usability and usefulness of MSProDiscuss in facilitating a discussion on disease progression between HCPs and patients in routine practice. Each HCP was expected to fill 10-40 initial questionnaires, depending on the patient population or country size. After completing all individual questionnaires, the HCPs also completed a final questionnaire on their overall assessment of MSProDiscuss, taking into account all previous consultations. The final questionnaire included 13 questions. In addition to those items covered in the initial questionnaire, we gathered feedback on the integration of MSProDiscuss into clinical practice. This two-step process was intended to ensure that the final feedback on usability and usefulness was received only after the HCPs had sufficient experience with the tool itself. The HCPs were also requested to provide general feedback on the features and performance of the tool in free-text fields.

### Summary Statistics

The HCP responses to the 2 usability questionnaires were analyzed separately with regard to the individual and final questionnaires and descriptively on a question-by-question basis and reported as a proportion of the total responses. The differences in the responses to the questionnaires by country were summarized by region and visualized as a heat map. No statistical comparisons were performed. To ensure that the survey sample was representative of the true population, both weighted and unweighted percentages were calculated. The weighted analysis ensured that the results were adjusted to reflect the underlying sample distribution. Therefore, weighting was performed at the country level with regard to the survey sample size. For the individual questionnaire, this refers to the number of patients for whom MSProDiscuss was used, whereas for the final questionnaire, the number of HCPs who participated in the survey was relevant (Multimedia Appendix 2). The feedback provided as free text was analyzed qualitatively by identifying common themes and categorizing them accordingly.

## Results

### Survey Participant Characteristics

**Figure 2:**
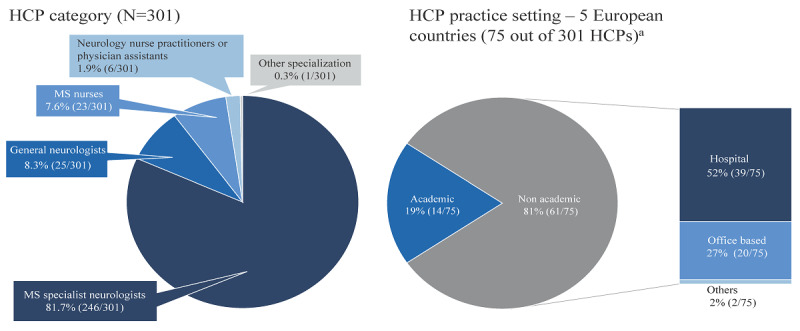
The characteristics of the survey participants. France, Germany, Italy, Spain, and the United Kingdom^a^. HCP: health care professional; MS: multiple sclerosis.

Of the 390 HCPs who were invited, 301 provided feedback on at least one questionnaire. Most of the participants were MS specialists (246/301, 81.7%), but also included general neurologists, MS nurses and nurse practitioners, and physician assistants (Figure 2). In a subanalysis of the participating HCPs from 5 countries in Europe (France, Germany, Italy, Spain, and the United Kingdom, which together represented 24.9% (75/301) of the participating HCPs), 52% (36/75) of the HCPs were from a hospital-based practice setting (Figure 2). Overall, the tool was assessed in different practice settings, ranging from academic hospitals to general hospitals and office-based practices.

Of the 301 HCPs who participated, 232 completed the expected number of questionnaires. During the MSProDiscuss usability test on a total of 6974 patients, the HCPs identified 5370 (77%) patients with RRMS, which also included those who may be transitioning to SPMS. The number of patients per HCP and the total number of individual consultations in the 5 European countries are shown in Table 1. Details from the regions are provided in Multimedia Appendix 3.

**Table 1:** MSProDiscuss^a^ use for the usability test in 5 European countries.

Country	HCPs^b^ who used the tool (n=75), n (%)	Patients diagnosed with SPMS^c^ at the time of the consultation during usability testing (n=477^d^), n (%)	Patients diagnosed with RRMS^e^ at the time of the consultation during usability testing (n=1513), n (%)	Number of times the tool was used in a consultation (n=1990), n (%)
France	9 (12)	5 (1)	11 (0.7)	16 (0.8)
Germany	27 (36)	175 (36.7)	542 (35.8)	717 (36)
Italy	14 (18.7)	167 (35)	357 (23.6)	524 (26.3)
Spain	19 (25.3)	121 (25.4)	583 (38.5)	704 (35.4)
United Kingdom	6 (8)	9 (1.9)	20 (1.3)	29 (1.5)

^a^MSProDiscuss: Multiple Sclerosis Progression Discussion Tool.

^b^HCP: health care professional.

^c^SPMS: secondary progressive multiple sclerosis.

^d^In this cohort of 5 European countries, 23.97% (477/1990) and 76.03% (1513/1990) of the patients were identified as patients with secondary progressive multiple sclerosis and patients with relapsing-remitting multiple sclerosis, respectively.

^e^RRMS: relapsing-remitting multiple sclerosis.

### Feedback on the Usability and Usefulness of MSProDiscuss

**Figure 3:**
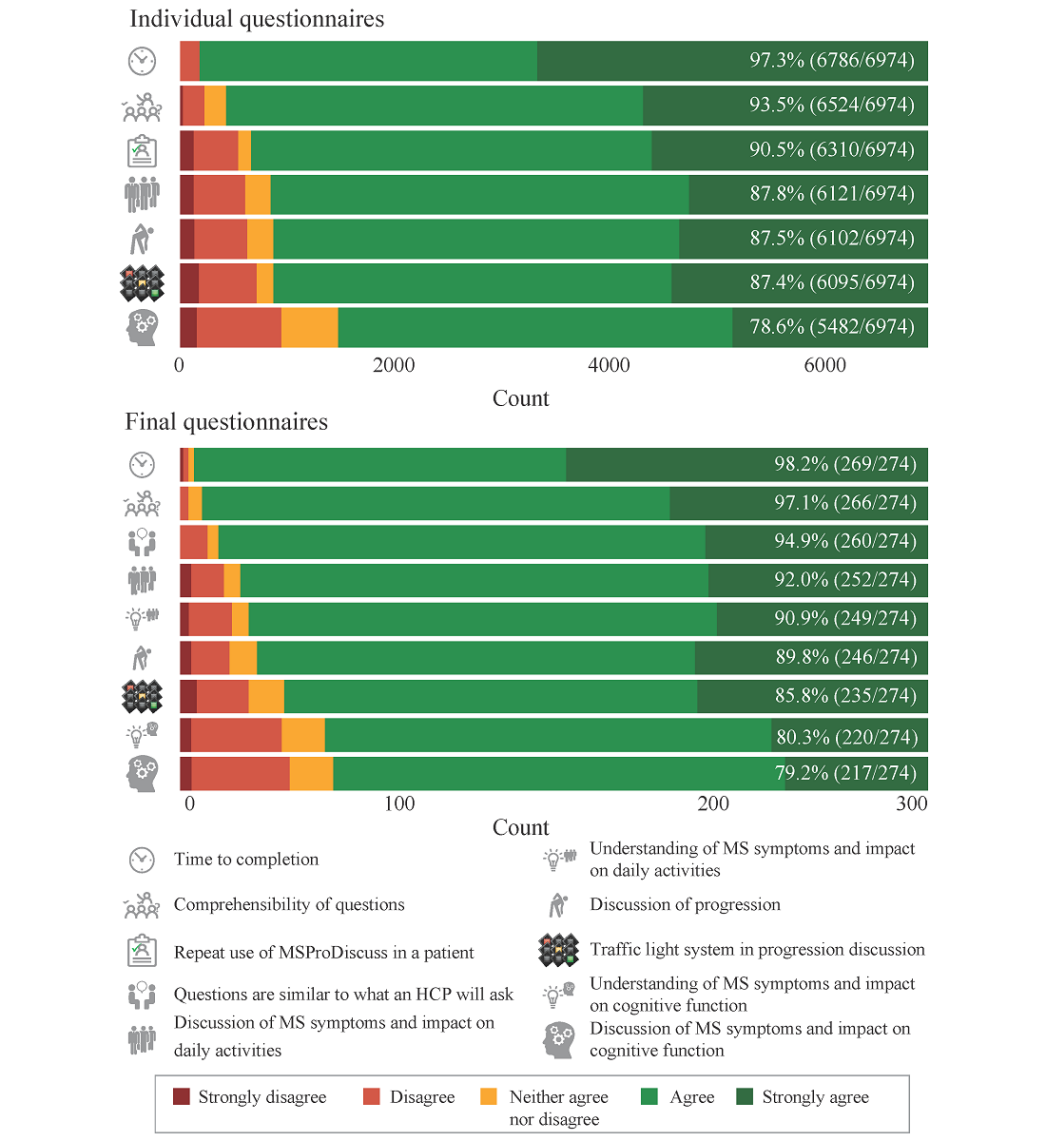
The summary findings from the usability and usefulness testing of the MSProDiscuss individual questionnaire (N=6974) and final questionnaire (N=274). The numbers in the bars reflect the proportion of health care professionals who responded agree or strongly agree for each item. Percentages are based on unweighted results; weighted results were similar. HCP: health care professional; MS: multiple sclerosis; MSProDiscuss: Multiple Sclerosis Progression Discussion Tool.

The usability and usefulness of the tool were assessed using both the initial and final questionnaires (Figure 3). The HCPs first completed individual questionnaires after using MSProDiscuss on 6974 people with MS and then completed a final questionnaire (N=274). MSProDiscuss was confirmed to be useful in relation to all the dimensions assessed. Most of the HCPs agreed or strongly agreed (217/274, 79.2%-269/274, 98.2%) that MSProDiscuss is beneficial in their practice (Figure 3). The time taken to complete the tool during a routine consultation was considered satisfactory (1-4 minutes) in 97.3% (6786/6974) of the initial questionnaires and in 98.2% (269/274) of the final questionnaires. The patients were able to comprehend the questions in the tool in) 93.5% (6524/6974) and 97.1% (266/274) of the cases (individual and final questionnaires, respectively). In 90.5% (6310/6974) of the individual questionnaires, the HCPs were willing to use the tool again with the same patient. MSProDiscuss was also useful in discussing MS symptoms and their impact on daily activities (6121/6974, 87.77% of the individual questionnaires and 252/274, 91.9% of the final questionnaires) and cognitive function (5482/6974, 78.61% for individual and 271/274, 79.2% for final questionnaires), as well as in discussing progression in general (6102/6974, 87.49% and 246/274, 89.8%, respectively). To summarize, there was excellent agreement between the initial and final questionnaires on all items and dimensions of usability and usefulness of the tool. Excellent usability and usefulness were reported from the initial instances, increasing slightly with repeated use.

The final questionnaire assessed additional usability aspects from the perspective of the HCPs. There was general agreement (260/274, 94.9% of the HCPs) that the questions in the tool were similar to those asked by an HCP during a regular consultation. MSProDiscuss was also found to be helpful in understanding the impact of MS symptoms on daily activities (249/274, 90.9% of the HCPs) and cognitive function (220/274, 80.3% of the HCPs). The findings based on the weighted analysis were similar to those based on the unweighted results (Multimedia Appendices 4 and 5). Overall, the tool was confirmed to be highly usable and useful in clinical practice.

**Figure 4:**
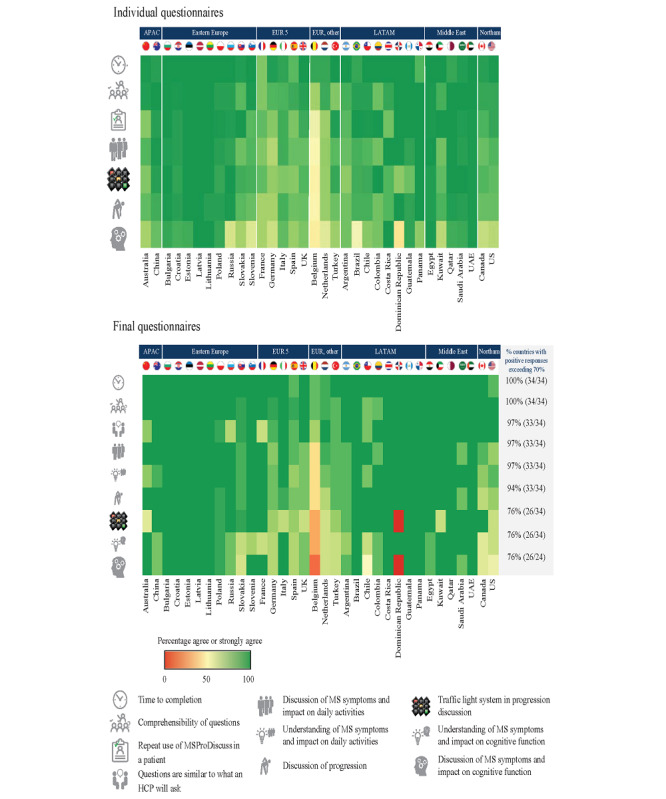
Distribution of responses on the usability and usefulness of MSProDiscuss by country and region: individual questionnaire (N=6974) and final questionnaire (N=274). APAC: Asia Pacific; EUR: Europe; HCP: health care professional; LATAM: Latin America; MS: multiple sclerosis; MSProDiscuss: Multiple Sclerosis Progression Discussion Tool; Northam: North America; UAE: United Arab Emirates.

On the individual questionnaires, the responses from the individual countries and regions were in line with the overall results, with the exception of Belgium, as shown in Figure 4. Satisfaction with the time taken to complete the tool (30/34, 88% of the countries), comprehensibility of the questions (25/34, 76% of the countries), and equivalence to routine questions asked by an HCP (24/34, 71% of the countries) were the top-scoring dimensions, with unanimous agreement across countries and regions.

### Feedback on Integration of MSProDiscuss Into Clinical Practice

**Figure 5:**
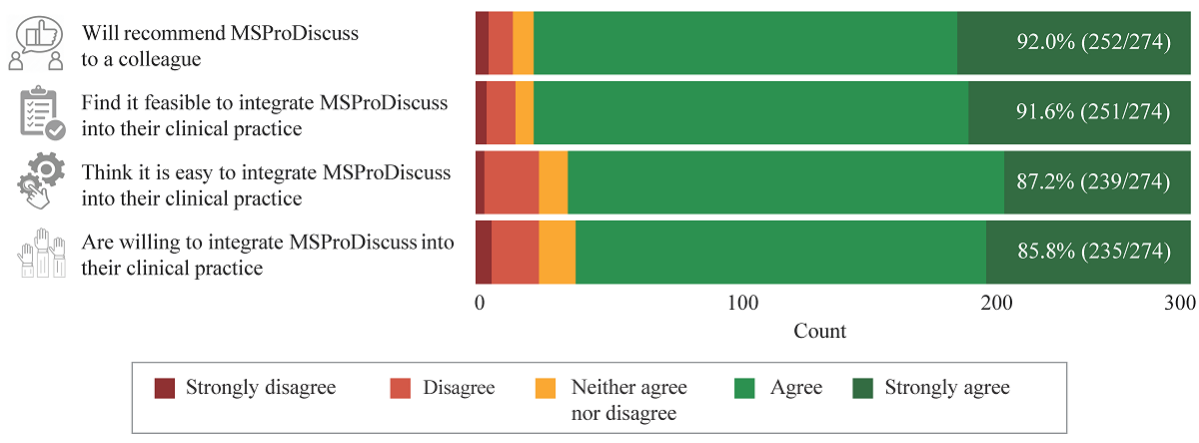
Integration of MSProDiscuss into clinical practice: summary findings from the final questionnaire (N=274). The numbers in the bars reflect the proportion of health care professionals who responded agree or strongly agree for each item. Percentages are based on unweighted results; weighted results are similar. MSProDiscuss: Multiple Sclerosis Progression Discussion Tool.

The final questionnaire included items to assess the adaptability of MSProDiscuss and the integration of the tool into routine clinical practice. The responses were very positive. Overall, 91.6% (251/274) of the HCPs thought that adaptability and integration are feasible, 87.2% (239/274) thought that adaptability and integration would be easy, and 85.8% (235/274) were willing to integrate MSProDiscuss into their clinical practice (Figure 5). Finally, 91.9% (252/274) of the HCPs would recommend MSProDiscuss to a colleague.

**Figure 6:**
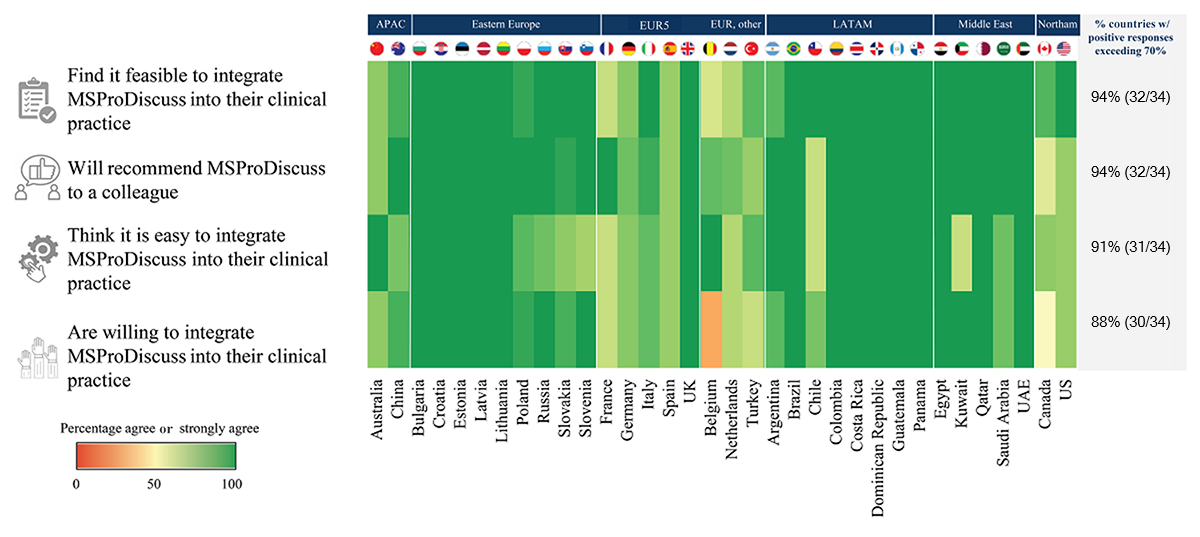
Distribution of health care professionals’ responses on the final questionnaire (N=274) by country (N=34) and region (N=7). APAC: Asia Pacific; EUR: Europe; LATAM: Latin America; MSProDiscuss: Multiple Sclerosis Progression Discussion Tool; Northam: North America; UAE: United Arab Emirates.

The findings at the country and regional levels were reflective of the overall results, with HCPs having high levels of agreement on the ease of implementation of the tool (Figure 6). An average agreement of more than 80% was seen in 76% (26/34) of countries and of more than 90% in 62% (21/34) of countries. In 53% (18/34) of countries, the HCPs unanimously agreed (ie, 100% in all 4 questions) that it is feasible and easy to integrate MSProDiscuss into their clinical practice, that they are willing to do so, and that they would recommend the tool to a colleague. This includes the United Kingdom and approximately half of the participating countries from Eastern Europe, Latin America, and the Middle East (Figure 6). Among the European countries, Belgium was an exception with a lower percentage of HCPs indicating willingness to integrate MSProDiscuss into clinical practice.

### Additional Feedback on MSProDiscuss

Throughout the development of MSProDiscuss, qualitative feedback was gathered, and improvements were implemented iteratively. A meaningful impact of this approach may be seen in the overall positive experience expressed by the HCPs while using MSProDiscuss. Of the 301 HCPs who returned the final questionnaire, 162 made at least one recommendation on improvements, such as including additional variables or expanding the existing variables. Table 2 lists these suggestions by topic and implementation status. Even before the conclusion of the survey on usability, implementation was already ongoing for several recommendations, including (1) integration of MSProDiscuss into electronic health record systems to allow for longitudinal patient follow-up in countries such as the United States, United Kingdom, and Germany; (2) an enhanced user interface for easier navigation; (3) improvement of the sensitivity of the tool for patients with lower Expanded Disability Status Scale (EDSS) scores or confounding symptoms such as fatigue; and, most importantly, (4) development of a patient version of the tool. Some of the other recommendations such as including cognitive or patient-reported outcomes measures are being considered for future updates of the tool. They need to be carefully weighed against the current high ease of use. Overall, the recommendations were focused on expanding the scope and reach of the tool, rather than improving basic usability, reinforcing the earlier conclusion that the tool is practical and easy to use.

**Table 2:** Recommendations from health care professionals on improvements.

Response	Number of responses	Details of the recommendation	Recommendation already implemented during usability testing
Expand components included in the tool or include additional questions	162	Add disease duration; add treatment adherence	N/A^a^
Interpretation of the traffic light output	79	Elaborate on the explanation or quantify the output	N/A
Improve or expand on the cognitive assessment	69	Include an interface with cognitive assessment scales	N/A
Include additional variables	57	Add more details on the impact of disease on daily activities, for example, relationships, social, work, sexuality, and emotional state	N/A
Improve tool sensitivity	56	Improve sensitivity of the tool	Improved sensitivity for patients with lower EDSS^b^ scores and with confounding symptoms such as fatigue
Include longitudinal follow-up	29	N/A	Included in observational studies for longitudinal monitoring; incorporated in electronic health records
Improve the user interface	29	N/A	User interface was improved on an ongoing basis
Provide a patient version	18	N/A	Development was ongoing in parallel to the usability testing. Meanwhile, the Your MS^c^ questionnaire is ready

^a^N/A: not applicable or implemented.

^b^EDSS: Expanded Disability Status Scale.

^c^MS: multiple sclerosis.

## Discussion

### SPMS Remains a Diagnostic Challenge

The highly variable disease course in individual patients and the lack of consensus on the diagnostic criteria often result in considerable delays in SPMS diagnosis [5,14]. In 2014, the study by Lublin et al [15] reported revised clinical and imaging findings to define clinical phenotypes. However, the study also acknowledged that objective criteria for separating clinical phenotypes are still lacking. There are no clear definitions of the transition from RRMS to SPMS. Until recently, no therapy with proven efficacy was available for a broad range of patients with SPMS. Thus, the diagnosis of SPMS is often delayed to avoid losing treatment options and reimbursement. With the advent of newer treatments for patients with SPMS, tools such as MSProDiscuss could be useful in supporting *real-time* evaluation of early signs of MS progression in routine clinical practice [16]. Tools based on algorithms and nomograms have been developed that use quantitative, data-driven empirical assessments. Although these tools can estimate the future risk of SPMS progression, they cannot easily be translated into current decisions in routine patient management [17-19]. MSProDiscuss provides additional insight through qualitative assessment of disease symptoms and their impact on daily life, thereby including, for the first time, the patient’s perspective in the overall shared decision-making. Timely diagnosis will allow for appropriate treatment and better long-term prognosis [14].

### Principal Findings

The results of this survey indicate that MSProDiscuss is a useful tool to aid the discussion of disease progression with patients. The findings were consistent between the individual and final questionnaires. Most of the HCPs agreed or strongly agreed that MSProDiscuss was beneficial in their practice. The time taken to complete the tool during routine consultation was considered satisfactory (1-4 minutes). In most instances, the questions in MSProDiscuss were found to be comprehensible by the patients and were similar to those asked by an HCP during a regular consultation. Thus, MSProDiscuss facilitates patient-physician discussion by capturing a structured disease history without imposing an additional time burden on the HCP. Furthermore, these positive results indicate that MSProDiscuss is easy to use and universally helpful, regardless of region, professional background, and practice setting.

More than 90.48% (6310/6974) of instances the HCPs indicated that they would use MSProDiscuss again with the same patients, including both patients with RRMS (4985/6310, 79%) and patients with SPMS (1325/6310, 20.99%). In particular, the HCPs indicated that they would use the tool again with more than 97% (5209/5370) of the patients with RRMS and with 94.01% (1508/1604) of the patients with SPMS. This indicates a potential role of MSProDiscuss as a complementary disease-monitoring tool for longitudinal follow-up to be added to current empirical measures such as clinical relapses or magnetic resonance imaging lesion counts. Qualitative insight into the symptoms and their impact on patients as captured by MSProDiscuss will help identify early signs of disease progression. In patients with RRMS, the tool might complement clinical assessments and help in treatment decision-making during the earliest stages of SPMS. For patients already diagnosed with SPMS, the tool will be helpful in identifying functional domains that are most affected by progression and in choosing the appropriate modalities for symptom management.

In this survey, 80.3% (220/274) or more of the HCPs indicated that MSProDiscuss is a useful aid in discussing MS symptoms and their impact on daily activities and cognitive function, as well as in discussing progression in general. The fact that 91.9% (252/274) of the HCPs would recommend MSProDiscuss to their colleagues and think that it is feasible to integrate MSProDiscuss into their clinical practice is very encouraging because it suggests that the tool is beneficial to patients as well as HCPs themselves. Key recommendations have already been implemented, including longitudinal follow-up, an enhanced user interface, improved sensitivity at lower EDSS scores, correction for overlapping fatigue symptoms, and creation of a patient-completed version. The suggestion to expand cognitive assessments is a key point; cognitive impairment is an important yet underrecognized sign of disease progression [20,21]. How empirical measures of cognitive performance could be integrated into this tool, without affecting the overall performance of the tool in terms of the time taken and ease of use during regular consultations, is an important goal for future development. Similarly, whether objective patient-reported outcomes measures could be integrated into MSProDiscuss remains to be evaluated.

Along with the traffic light output, considering the patient’s responses to the individual questions provides additional insights, supporting the relevance of MSProDiscuss for holistic disease management in individual patients. MSProDiscuss is suitable for longitudinal follow-up and has already been included in large observational studies [22] and integrated into the electronic health records in several countries. This will ensure the systematic recording of a patient’s disease evolution and individual patient monitoring over time. The tool will assist in defining a multidisciplinary treatment strategy for individual patients, including physiotherapy, rehabilitation, and relevant symptomatic treatments. As MSProDiscuss covers several functional domains, it provides comprehensive information on the patient’s health status, including MS phenotype and symptomatology [23]. Although EDSS assessment is viewed as the gold standard for measuring physical disability, it has been implemented in clinical routine on a limited scale [24-26]. MSProDiscuss will complement EDSS assessment in routine practice by capturing symptoms and their impact between consecutive EDSS assessments.

A patient version of the tool, the Your MS questionnaire, was developed to cover patient-derived information on MS symptoms also contained in MSProDiscuss (Multimedia Appendix 6). The Your MS questionnaire can be completed by the patient in preparation for a clinical visit, potentially with help from a caregiver who can provide additional information [27]. The Your MS questionnaire may further complement patient-physician interactions on disease progression and is expected to not only help reduce the on-call burden on physicians without compromising on the quality of consultation, but also positively involve patients in the management of their own disease. The use of telemedicine is an increasing trend in the management of chronic diseases [28,29]; most recently, the implementation of telemedicine in the management of visits by patients with MS has been accelerated by the COVID-19 pandemic [30,31]. In the context of teleconsultation, the Digital Technologies Web and Social Media Study Group recently suggested a battery for assessing MS disability and relapse, proposing commonly used tools that are suitable before, during, and after a teleconsultation [32]. Being a web-based tool, MSProDiscuss was tested during the COVID-19 pandemic, and it showed promise in assisting with remote visits where the lack of face-to-face interaction can hamper communication. As one of the first tools of its kind, MSProDiscuss is able to assist with deep clinical phenotyping of signs of progression, based on the physician’s documented patient history [33]. As a tool with promise, the patient-completed Your MS questionnaire can either be used ahead of traditional face-to-face visits, or it may be integrated into the suggested battery in preparation for a teleconsultation. Although implementation of these digital or web-based tools in the management of MS is a necessity during the pandemic, these tools are unlikely to fully replace face-to-face consultations [30]. In-person evaluation and differential diagnosis of MS symptoms remain vital, and especially in light of technological and psychological limitations, web-based tools will remain complementary to in-person consultations [34].

Overall, the results were consistent at the individual country and region levels, with some minor variations reflective of either individual HCP preference of disease management or general practice guidelines followed in the country. The overall positive feedback from most of the countries and regions suggests that MSProDiscuss is already perceived as a valuable tool. In some countries such as Belgium where the feedback was less encouraging in terms of integration into clinical practice, a root cause analysis could not be performed because of the limitations of the study design. It can be speculated that, with limited treatment options in some geographies, it is likely that there is caution regarding discussing disease progression when no solution can be proposed. In other cases, there may be reluctance to discuss disease progression because of the potential emotional and psychological implications for the patient. It is likely that these factors could have influenced the respondents in our survey. However, this only re-emphasizes the need for clear and transparent discussions regarding the importance of identifying early signs of disease progression, allowing treatments and holistic modalities to be started sooner.

### Study Limitations

Gathering feedback on the usability and usefulness of the tool while simultaneously using the tool might have resulted in a potential bias in the HCPs’ responses to certain components of the survey, such as integration into practice and time taken for completion. In general, the questionnaire methodology only highlights trends or attitudes and does not explain the underlying reasons for the responses [35].

### Outlook

Overall, the positive findings from our usability and usefulness study of MSProDiscuss are very promising [36]. Although centers with a heavy patient inflow might find it difficult to implement a new tool in their workflow, our results show that HCPs across different practice settings can easily integrate MSProDiscuss into their routine practice. MSProDiscuss does not require extensive data to be collected or curated to assess the level of disease progression, and it is complementary to other approaches based on imaging, neurological examination, or biomarkers. As a valuable disease-monitoring tool, advantages in the long term should outweigh the initial implementation challenges, if any. When used together with Your MS, the patient version, MSProDiscuss can also be a valuable tool in the day-to-day management of people with MS through telemedicine, even during crisis situations. The future impact on long-term disease monitoring and health care resource utilization remains to be evaluated.

### Conclusions

The findings from this real-world study suggest that MSProDiscuss is a usable and useful tool to facilitate a physician-patient discussion on disease progression in daily clinical practice. MSProDiscuss facilitates the dialog between the patient and the physician by capturing a structured disease history. Most of the survey participants indicated that MSProDiscuss was beneficial in the discussion of disease progression. Overall, the feedback from the HCPs was very positive regarding the integration of MSProDiscuss into their clinical practice. The tool was used by physicians, MS nurses and nurse practitioners, and pharmacists from very different practice settings and was found to be of value; MSProDiscuss is a tool that is acceptable to all the users involved in the care and management of patients with MS.

## Appendix

Multimedia Appendix 1 Usability test questions (responses to each question were provided on a 5-point Likert scale).

Multimedia Appendix 2 Illustration showing the principle of weighting.

Multimedia Appendix 3 Multiple Sclerosis Progression Discussion Tool use for the usability test by region and patient type.

Multimedia Appendix 4 Summary findings from the individual questionnaire: weighted results.

Multimedia Appendix 5 Summary findings from the final questionnaire: weighted results.

Multimedia Appendix 6 Screenshots of the Your MS Questionnaire.

## Abbreviations

EDSS: Expanded Disability Status Scale
HCP: health care professional
MS: multiple sclerosis
MSProDiscuss: Multiple Sclerosis Progression Discussion Tool
RRMS: relapsing-remitting multiple sclerosis
SPMS: secondary progressive multiple sclerosis
