# Supplementary material for: Multiple Sclerosis Progression Discussion Tool Usability and Usefulness in Clinical Practice: Cross-sectional, Web-Based Survey
Source: J Med Internet Res. 2021 Oct 6;23(10):e29558. doi: 10.2196/29558 (PMC8529467; doi:10.2196/29558)
Supplement: Multimedia Appendix 1 [file jmir_v23i10e29558_app1.docx]

## **Multimedia Appendix 1**

Table S1. Usability test questions (responses to each question were provided on a 5-point Likert scale)

|  | **Initial questionnaire** | **Final questionnaire** |
| --- | --- | --- |
| Questions related to usability and comprehensibility | 1. The time it took me to complete the tool was satisfactory. | 1. The time it took me to complete the tool was satisfactory. |
|  | 2. The patient was able to comprehend the questions from the tool that I asked in the course of the patient consultation. | 1. The patients were able to comprehend my questions from the tool during consultations. |
|  | 3. I would use this tool again in the future with this patient. | 1. The questions in the tool are similar to what I would ask during a regular consultation. |
| Questions related to usefulness | 4. The tool helped me discuss symptoms of MS progression and the impact on patients’ daily activities. | 1. The tool helped me better discuss symptoms suggestive of MS progression and its impact on patients’ daily activities. |
|  |  | 1. The tool helped me better understand symptoms suggestive of MS progression and its impact on patients’ daily activities. |
|  | 5. The tool helped me discuss the topic of progression with my patient. | 1. The tool helped me discuss the topic of progression with my patients. |
|  | 6. The green/yellow/red results at the end of the questionnaire helped me discuss progression with my patient. | 1. The green/yellow/red results at the end of the questionnaire helped me discuss progression with my patients. |
|  | 7. The tool helped me discuss symptoms of MS progression and its impact on cognitive function. | 1. The tool helped me better discuss symptoms suggestive of MS progression and its impact on cognitive function. |
|  |  | 1. The tool helped me better understand symptoms suggestive of MS progression and its impact on cognitive function. |
| Questions related to integration to clinical practice |  | 1. I would recommend this tool to a colleague. |
|  |  | 1. It is feasible to integrate the tool into my clinical practice. |
|  |  | 1. It would be easy to integrate the tool into my clinical practice. |
|  |  | 1. I would be willing to integrate the tool into my clinical practice. |

MS, multiple sclerosis
