# Supplementary material for: Multiple Sclerosis Progression Discussion Tool Usability and Usefulness in Clinical Practice: Cross-sectional, Web-Based Survey
Source: J Med Internet Res. 2021 Oct 6;23(10):e29558. doi: 10.2196/29558 (PMC8529467; doi:10.2196/29558)
Supplement: Multimedia Appendix 3 [file jmir_v23i10e29558_app3.docx]

## **Multimedia Appendix 3**

MSProDiscuss use for the usability test by region and patient type

| **Region** | **Country** | **Number of patients diagnosed with SPMS at the time of the consultation during usability testing** | **Number of patients diagnosed with RRMS at the time of the consultation during usability testing** | | | **Number of HCPs who used the tool** | **Number of times the tool was used in consultation** |
| --- | --- | --- | --- | --- | --- | --- | --- |
| **APAC** | Australia | 57 | | 118 | 9 | | 175 |
|  | China | 17 | | 91 | 10 | | 108 |
| **Eastern Europe** | Bulgaria | 19 | | 253 | 13 | | 272 |
|  | Croatia | 25 | | 132 | 4 | | 157 |
|  | Estonia | 5 | | 37 | 2 | | 42 |
|  | Latvia | 2 | | 39 | 2 | | 41 |
|  | Lithuania | 1 | | 60 | 3 | | 61 |
|  | Poland | 143 | | 499 | 18 | | 642 |
|  | Russia | 48 | | 97 | 8 | | 145 |
|  | Slovakia | 31 | | 37 | 25 | | 68 |
|  | Slovenia | 44 | | 116 | 4 | | 160 |
| Europe – 5 countries | France | 5 | | 11 | 9 | | 16 |
|  | Germany | 175 | | 542 | 27 | | 717 |
|  | Italy | 167 | | 357 | 14 | | 524 |
|  | Spain | 121 | | 583 | 19 | | 704 |
|  | UK | 9 | | 20 | 6 | | 29 |
| Europe – Others | Belgium | 43 | | 162 | 10 | | 205 |
|  | Netherlands | 109 | | 267 | 15 | | 376 |
|  | Turkey | 64 | | 269 | 12 | | 333 |
| Middle East | Egypt | 48 | | 278 | 18 | | 326 |
|  | Kuwait | 21 | | 70 | 3 | | 91 |
|  | Qatar | 3 | | 37 | 1 | | 40 |
|  | Saudi Arabia | 16 | | 84 | 7 | | 100 |
|  | UAE | 32 | | 171 | 6 | | 203 |
| LATAM | Argentina | 45 | | 168 | 10 | | 213 |
|  | Brazil | 13 | | 43 | 2 | | 56 |
|  | Chile | 45 | | 233 | 8 | | 278 |
|  | Colombia | 30 | | 125 | 8 | | 155 |
|  | Costa Rica | 4 | | 16 | 1 | | 20 |
|  | Dominican Republic | 1 | | 9 | 1 | | 10 |
|  | Guatemala | 3 | | 18 | 2 | | 21 |
|  | Panama | 0 | | 10 | 1 | | 10 |
| North America | Canada | 161 | | 248 | 12 | | 409 |
|  | US | 64 | | 203 | 11 | | 267 |

APAC, Asia Pacific; 5 countries; HCP, healthcare professionals; LATAM, Latin America; MSProDiscuss: Multiple Sclerosis Progression Discussion Tool; RRMS, relapsing–remitting multiple sclerosis; SPMS, secondary progressive multiple sclerosis; UAE, United Arab Emirates; UK, United Kingdom; US, United States
