# Supplementary material for: Multiple Sclerosis Progression Discussion Tool Usability and Usefulness in Clinical Practice: Cross-sectional, Web-Based Survey
Source: J Med Internet Res. 2021 Oct 6;23(10):e29558. doi: 10.2196/29558 (PMC8529467; doi:10.2196/29558)
Supplement: Multimedia Appendix 4 [file jmir_v23i10e29558_app4.docx]

## **Multimedia Appendix 4**

Figure S1. Summary findings from the individual questionnaire: weighted results.


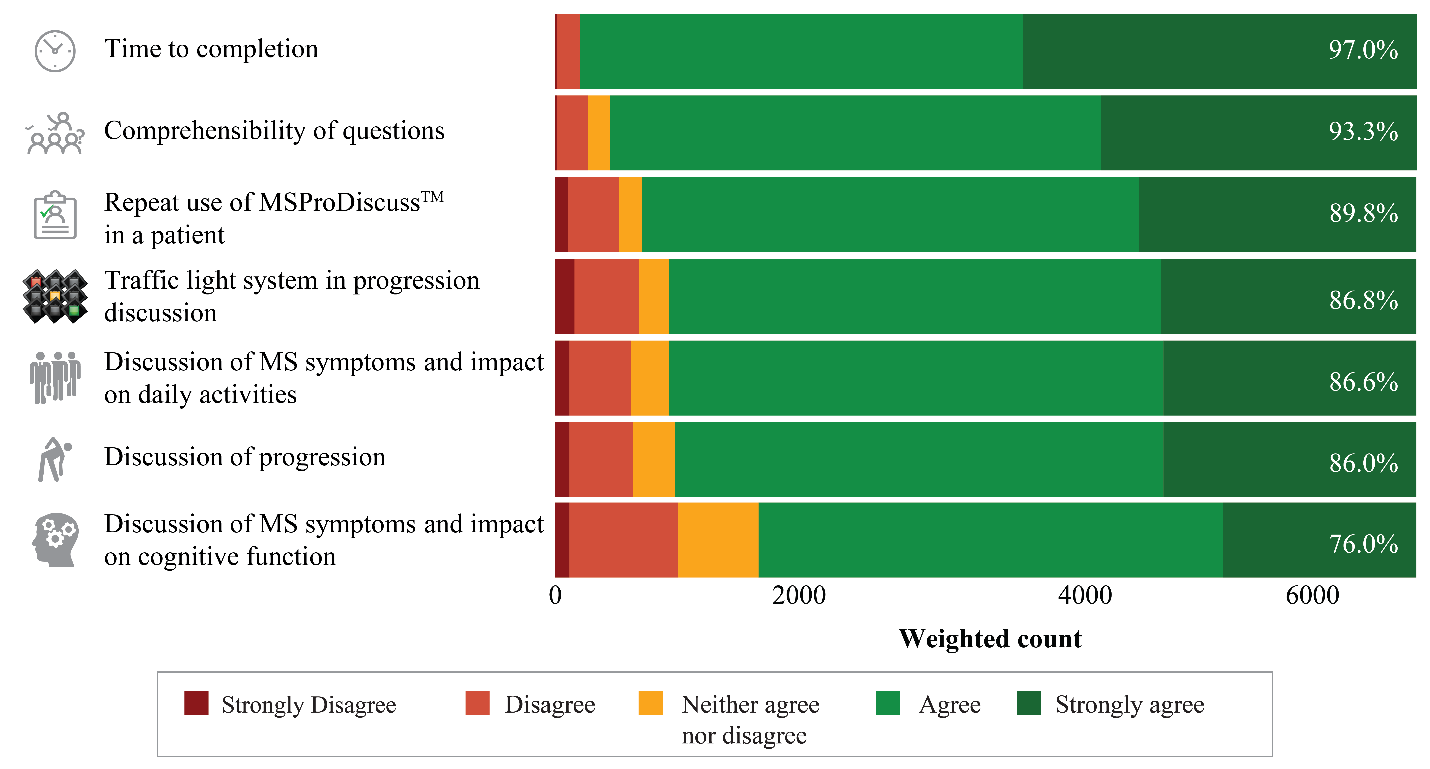


MSProDiscuss: Multiple Sclerosis Progression Discussion Tool.

Table S1. Weights for individual questionnaires.

| **Country** | **No. of individual responses** | **No. of MS patients (2016)^a^** | **Population size (millions)** | **MS patients per million** | **Representation in sample (%)** | **Representation in population (%)** | **Country weight** |
| --- | --- | --- | --- | --- | --- | --- | --- |
| Argentina | 213 | 20248 | 44.27 | 457 | 3.1 | 1.9 | 0.6 |
| Australia | 175 | 22298 | 24.6 | 906 | 2.5 | 3.9 | 1.5 |
| Belgium | 205 | 14752 | 11.4 | 1294 | 2.9 | 5.5 | 1.9 |
| Brazil | 56 | 29467 | 209.3 | 141 | 0.8 | 0.6 | 0.7 |
| Bulgaria | 272 | 3115 | 7.05 | 442 | 3.9 | 1.9 | 0.5 |
| Canada | 409 | 79419 | 37.59 | 2113 | 5.9 | 9 | 1.5 |
| Chile | 278 | 9362 | 18.05 | 519 | 4 | 2.2 | 0.6 |
| China | 108 | 103194 | 1386 | 74 | 1.5 | 0.3 | 0.2 |
| Colombia | 155 | 2662 | 49.07 | 54 | 2.2 | 0.2 | 0.1 |
| Costa Rica | 20 | 355 | 4.906 | 72 | 0.3 | 0.3 | 1.1 |
| Croatia | 157 | 2019 | 4.076 | 495 | 2.3 | 2.1 | 0.9 |
| Dominican Republic | 10 | 1031 | 10.77 | 96 | 0.1 | 0.4 | 2.8 |
| Egypt | 326 | 29566 | 97.55 | 303 | 4.7 | 1.3 | 0.3 |
| Estonia | 42 | 913 | 1.325 | 689 | 0.6 | 2.9 | 4.9 |
| France | 16 | 65467 | 66.99 | 977 | 0.2 | 4.2 | 18.1 |
| Germany | 717 | 111970 | 82.79 | 1352 | 10.3 | 5.8 | 0.6 |
| Guatemala | 21 | 846 | 16.91 | 50 | 0.3 | 0.2 | 0.7 |
| Italy | 524 | 72352 | 60.48 | 1196 | 7.5 | 5.1 | 0.7 |
| Kuwait | 91 | 2039 | 4.137 | 493 | 1.3 | 2.1 | 1.6 |
| Latvia | 41 | 1303 | 1.92 | 679 | 0.6 | 2.9 | 4.9 |
| Lithuania | 61 | 1852 | 2.794 | 663 | 0.9 | 2.8 | 3.2 |
| Netherlands | 376 | 25197 | 17.18 | 1467 | 5.4 | 6.2 | 1.2 |
| Panama | 10 | 255 | 4.099 | 62 | 0.1 | 0.3 | 1.8 |
| Poland | 642 | 36049 | 37.98 | 949 | 9.2 | 4 | 0.4 |
| Qatar | 40 | 862 | 2.639 | 327 | 0.6 | 1.4 | 2.4 |
| Russia | 145 | 93975 | 144.5 | 650 | 2.1 | 2.8 | 1.3 |
| Saudi Arabia | 100 | 10840 | 32.94 | 329 | 1.4 | 1.4 | 1 |
| Slovakia | 68 | 3372 | 5.45 | 619 | 1 | 2.6 | 2.7 |
| Slovenia |  | 1487 | 2.084 | 714 | 2.3 | 3 | 1.3 |
| Spain | 704 | 43867 | 46.66 | 940 | 10.1 | 4 | 0.4 |
| Turkey | 333 | 61408 | 80.81 | 760 | 4.8 | 3.2 | 0.7 |
| UAE | 203 | 4216 | 9.4 | 449 | 2.9 | 1.9 | 0.7 |
| UK | 29 | 106454 | 66.44 | 1602 | 0.4 | 6.8 | 16.4 |
| US | 267 | 511855 | 327.2 | 1564 | 3.8 | 6.7 | 1.7 |

^a^Figures obtained from the Global, regional, and national burden of multiple sclerosis study, Lancet Neurol 2019; 18: 269–85 MS, multiple sclerosis; UAE, United Arab Emirates; UK, United Kingdom; US, United States
