# Supplementary material for: Multiple Sclerosis Progression Discussion Tool Usability and Usefulness in Clinical Practice: Cross-sectional, Web-Based Survey
Source: J Med Internet Res. 2021 Oct 6;23(10):e29558. doi: 10.2196/29558 (PMC8529467; doi:10.2196/29558)
Supplement: Multimedia Appendix 5 [file jmir_v23i10e29558_app5.docx]

## **Multimedia Appendix 5**

Figure S2. Summary findings from the final questionnaire: weighted results.



MSProDiscuss: Multiple Sclerosis Progression Discussion Tool

Table S3. Weights for final questionnaires

| **Country** | **No. of participating**  **HCPs** | **Total no. of neurologists treating MS^a^** | **Representation in sample (%)** | **Representation in population (%)** | **Country weight** |
| --- | --- | --- | --- | --- | --- |
| Argentina | 10 | 100 | 3.3 | 0.8 | 0.3 |
| Australia | 9 | 100 | 3 | 0.8 | 0.3 |
| Belgium | 10 | 346 | 3.3 | 2.9 | 0.9 |
| Brazil | 2 | 250 | 0.7 | 2.1 | 3.2 |
| Bulgaria | 13 | 40 | 4.3 | 0.3 | 0.1 |
| Canada | 11 | 200 | 4 | 1.7 | 0.4 |
| Chile | 8 | 28 | 2.7 | 0.2 | 0.1 |
| China | 10 | 200 | 3.3 | 1.7 | 0.5 |
| Colombia | 8 | 32 | 2.7 | 0.3 | 0.1 |
| Costa Rica | 2 | 15 | 0.7 | 0.1 | 0.2 |
| Croatia | 4 | 31 | 1.3 | 0.3 | 0.2 |
| Dominican Republic | 1 | 15 | 0.3 | 0.1 | 0.4 |
| Egypt | 18 | 100 | 6 | 0.8 | 0.1 |
| Estonia | 10 | 300 | 0.7 | 2.5 | 3.8 |
| France | 9 | 210 | 3 | 1.8 | 0.6 |
| Germany | 26 | 3200 | 8.6 | 26.8 | 3.1 |
| Guatemala | 2 | 15 | 0.7 | 0.1 | 0.2 |
| Italy | 14 | 300 | 4.7 | 2.5 | 0.5 |
| Kuwait | 3 | 100 | 1 | 0.8 | 0.8 |
| Latvia | 2 | 300 | 0.7 | 2.5 | 3.8 |
| Lithuania | 3 | 300 | 1 | 2.5 | 2.5 |
| Netherlands | 15 | 110 | 5 | 0.9 | 0.2 |
| Panama | 1 | 15 | 0.3 | 0.1 | 0.4 |
| Poland | 18 | 100 | 6 | 0.8 | 0.1 |
| Qatar | 1 | 100 | 0.3 | 0.8 | 2.5 |
| Russia | 8 | 100 | 2.7 | 0.8 | 0.3 |
| Saudi Arabia | 7 | 100 | 2.3 | 0.8 | 0.4 |
| Slovakia | 25 | 24 | 8.3 | 0.2 | <0.1 |
| Slovenia | 4 | 30 | 1.3 | 0.3 | 0.2 |
| Spain | 19 | 100 | 6.3 | 0.8 | 0.1 |
| Turkey | 12 | 450 | 4 | 3.8 | 0.9 |
| UAE | 6 | 55 | 2 | 0.5 | 0.2 |
| UK | 6 | 1000 | 2 | 8.4 | 4.2 |
| US | 11 | 3560 | 3.7 | 29.9 | 8.2 |

^a^In case this was not provided for the country, the median value across countries was taken. In case of country groups, eg, Central America & the Caribbean, the total reported for the country group was divided across the constituent states.

HCP, healthcare professional; MS, multiple sclerosis; UAE, United Arab Emirates; UK, United Kingdom; US, United States
