# Supplementary material for: Multiple Sclerosis Progression Discussion Tool Usability and Usefulness in Clinical Practice: Cross-sectional, Web-Based Survey
Source: J Med Internet Res. 2021 Oct 6;23(10):e29558. doi: 10.2196/29558 (PMC8529467; doi:10.2196/29558)
Supplement: Multimedia Appendix 6 [file jmir_v23i10e29558_app6.docx]

## **Multimedia Appendix 6**

Screenshots of the Your MS Questionnaire.

MS: Multiple Sclerosis.
